# Descriptive epidemiology of changes in objectively measured sedentary behaviour and physical activity: six-year follow-up of the EPIC-Norfolk cohort

**DOI:** 10.1186/s12966-018-0746-5

**Published:** 2018-11-27

**Authors:** Samantha Hajna, Tom White, Søren Brage, Esther M. F. van Sluijs, Kate Westgate, Andy P. Jones, Robert Luben, Kay-Tee Khaw, Nicholas J. Wareham, Simon J. Griffin

**Affiliations:** 1 MRC Epidemiology Unit, University of Cambridge, Cambridge, UK; 2 Norwich Medical School, University of East Anglia, Norwich, UK; 3 Department of Public Health and Primary Care, University of Cambridge, Cambridge, UK

**Keywords:** Sedentary time, Physical activity, Accelerometry

## Abstract

**Background:**

Sedentary time increases and total physical activity decreases with age. The magnitude and correlates of changes in sedentary time, light-intensity physical activity (LPA), moderate-to-vigorous intensity physical activity (MVPA), and overall physical activity remain unclear. We quantified these changes and identified their individual and sociodemographic correlates.

**Methods:**

We used data from 1259 adults (67.8 ± 6.9 years; 41.9% women) who participated in the EPIC-Norfolk Study. Activity was assessed at baseline (2004–2011) and follow-up (2012–2016) for 7 days using accelerometers. Potential correlates of change were specified a priori. We used unadjusted and adjusted sex-stratified linear regressions to identify correlates of change.

**Results:**

Only 3.7% of adults met the current MVPA recommendations. Sedentary time increased by 3.0 min/day/year (SD = 12.3). LPA, MVPA, and overall PA decreased by 1.7 min/day/year (SD = 5.4), 3.0 min/day/year (SD = 6.0), and 8.8 cpm/year (SD = 18.8), respectively. Correlates of greater rates of increase in sedentary time included older age and higher BMI in men, and older age, higher BMI, smoking, and urban dwelling in women. Correlates of greater rates of decrease in physical activity included older age, higher BMI, living alone, depression, car use, and/or fair/poor self-rated health in men, and older age, higher BMI, depression, smoking, and/or urban dwelling in women (e.g. depressed women had a 1.0 min/day/year greater rate of decline in MVPA than non-depressed women, 95% CI -1.8, − 0.2).

**Conclusions:**

Most (> 95%) adults are insufficiently active. Sedentary time increases and LPA, MVPA and overall physical activity decreases over time, with more pronounced rates of change observed in specific sub-groups (e.g. among older and depressed adults). To promote active living, the correlates of these changes should be considered in future interventions.

**Electronic supplementary material:**

The online version of this article (10.1186/s12966-018-0746-5) contains supplementary material, which is available to authorized users.

## Background

Achieving sufficient levels of physical activity is associated with decreased risk of chronic diseases (e.g. diabetes, cancer, depression) and premature mortality [1]. It is also associated with decreased risk of dementia, greater mobility, improved quality of life, and greater independence in later life [2–4]. Despite these benefits, it is estimated that 27.5% of adults are insufficiently active and at risk for inactivity-related health complications [5].

To inform the development and targeting of interventions designed to mitigate negative changes in activity, researchers have sought to quantify temporal changes in activity [6–9] and to identify their correlates [8–12]. For example, in a recent 10-year follow-up analysis of 962 participants of the Coronary Artery Risk Development in Young Adults (CARDIA) study [6], sedentary time was found to increase by 37.9 min/day, and light-intensity physical activity (LPA), unbouted moderate-to-vigorous intensity physical activity (MVPA), and total activity were found to decrease by 30.6 min/day, 7.5 min/day and 65.5 cpm (17%) over a decade, respectively [6]. Changes in activity were patterned by race and sex [6]. While changes in physical activity and their correlates have been previously assessed, most studies, particularly those of older adults, have been restricted to the assessment of total physical activity volume [8, 9]. Since sedentary behaviours and light-intensity physical activity (LPA) have important implications for health [13–15], more research on temporal changes across all ranges of activity intensity are needed.

Sedentary behaviours are any waking behaviours that are characterised by an energy expenditure ≤1.5 metabolic-equivalent units (METs) and conducted in either a sitting, reclined, or lying down position (e.g. reading, watching television) [16]. LPA is any activity characterised by an energy expenditure between 1.5 and 3 METs and includes activities typical of routine daily activities (e.g. cooking, walking around the house) or low-intensity recreational and occupational activities (e.g. playing darts) [17]. Both lower levels of sedentary time and higher levels of LPA have been linked to decreased chronic disease risk [18–20]. Since decreasing sedentary time and increasing light-intensity activities may require only small changes to routine day-to-day activities, changing these activity intensities may be easier than increasing total activity volume or higher-intensity activity [13]. Quantifying changes in lower activity intensities and identifying the correlates of these changes may prove useful in identifying effective targets for intervention development and evaluation.

Our aim was to build on the existing literature base by estimating, in a population-based sample of middle-age and older English adults, six-year changes in sedentary time, light-intensity physical activity (LPA), moderate-to-vigorous intensity physical activity (MVPA), and overall physical activity using objective activity monitoring and to identify the individual and sociodemographic correlates of these changes.

## Methods

We used data collected as part of the European Prospective Investigation of Cancer (EPIC)-Norfolk Study. In brief, 25,639 participants were recruited from 35 general practices and invited to attend a clinic assessment between 1993 and 1997. Participants were invited for second, third, and fourth in-clinic assessments between 1998 and 2000, 2004 and 2011, 2012 and 2016, respectively. Physical activity was assessed using accelerometers in the third and fourth assessments. For the purposes of this paper we refer to these 3rd and 4th health checks as the baseline and follow-up visits, respectively.

### Sedentary time and physical activity

Participants wore an accelerometer (ActiGraph, Pensacola, FL) on their right hip for seven days excluding during bathing, swimming, or sleeping and returned their accelerometers to the research unit after the monitoring period using a postage paid envelope. Uniaxial accelerometers (GT1M; data recorded in five-second epochs) were worn at the baseline visit and triaxial accelerometers (GT3X+; data recorded at 100 Hz) were worn at the follow-up visit. Harmonization of the GT1M and the GT3X+ data involved converting GT3X+ acceleration to counts in five-second epochs with the low-frequency extension filter disabled to match the on-board filtering performed by the GT1M firmware and retaining only the vertical axis data collected by the triaxial GT3X+ monitor (which is the measured axis in the uniaxial GT1M accelerometer); this method produces virtually identical results during standardised movements [21]. In addition, strong agreement has been previously demonstrated between these two accelerometers in human experiments both in terms of activity volume and intensity making them acceptable for use in a single study [17].

Only participants with ≥4 valid days of data (weekend or weekday) at both baseline and follow-up were included in this study. A valid day was defined as having ≥600 min of wear time in a day, with non-wear time defined as time segments with ≥90 min of continuous zero activity counts [22].

A review of the activity plots suggested that participants who wore their accelerometers for ≥19 h/day wore their accelerometers while sleeping. Since wear time may be correlated with our activity measures of interest, we excluded these participants to ensure the accurate assessment of activity levels and we also adjusted for wear time at baseline and follow-up in our regression analyses. Sedentary time, LPA, and MVPA were expressed in minutes/day and were classified according to the following counts per minute (cpm) cut-offs: sedentary time (< 100 cpm), LPA (100 to 808 cpm), and MVPA (≥809 cpm) [22]. The latter cut-point is based on the lower bound of MVPA identified in a study of older adults performing walking-based activities [23]. Overall physical activity (i.e. mean cpm) was defined as total activity counts divided by valid wear time.

The World Health Organization recommends that adults accumulate at least 150 min per week of MVPA in bouts of at least 10 min [5, 24]. We chose unbouted MVPA (i.e. cumulative MPVA) as our primary MVPA variable of interest because it also has benefits for health [25, 26] and may be easier for adults to accumulate than bouted MVPA. Nevertheless, to allow for the evaluation of activity profiles in the context of the current MVPA recommendations, we also present some descriptive statistics for bouted MVPA and regression estimates for the correlates of changes in bouted MVPA as a supplement. Bouted MVPA (min/day) was calculated as activity at an intensity ≥809 cpm sustained for 10 min or more. Participants were classified as meeting MVPA guidelines if they accumulated ≥21.4 min/day of bouted MVPA (i.e. ≥150 min/week of MVPA).

### Individual and sociodemographic factors

We identified factors for inclusion into this study based on a priori evidence that they may be correlated with sedentary time and/or physical activity [11, 27–30] and thus, we hypothesized, may also be potentially important baseline predictors of changes in these activities over time. Table 1 includes a summary of the individual and sociodemographic factors that were assessed in this study. With the exception of body mass index (BMI; kg/m^2^), which was calculated based on height and weight measurements collected by trained research assistants, all of the individual and sociodemographic factors were assessed via a standardised questionnaire. Follow-up time was calculated as the difference in years between the baseline and follow-up visits.

**Table 1:** Characteristics of the EPIC-Norfolk study sample at baseline, overall and by sex

	Overall	Men	Women
	*n = 1259*	*n = 528*	*n = 731*
	mean (SD)		
Age, *years* (Range: 49 to 91 years)	67.8 (6.9)	68.9 (6.9)	66.9 (6.8)
Body mass index, *kg/m*^*2*^	26.4 (4.2)	26.7 (3.4)	26.2 (4.7)
	% (n)		
Education level^a^			
O-level or lower	34.4 (433)	27.7 (146)	39.3 (287)
A-level	47.2 (594)	52.4 (277)	43.4 (317)
Degree	18.4 (232)	19.9 (105)	17.4 (127)
Paid job at present			
Yes	28.4 (357)	31.8 (168)	25.9 (189)
No	71.6 (902)	68.2 (360)	74.1 (542)
Marital status			
Married/living with partner	83.1 (1046)	90.3 (477)	77.8 (569)
Single/widowed/separated/divorced	16.9 (213)	9.7 (51)	22.2 (162)
Self-reported depression requiring treatment			
Yes	21.7 (273)	14.4 (76)	26.9 (197)
No	78.3 (986)	85.6 (452)	73.1 (534)
Household dog ownership			
Yes	19.4 (244)	17.8 (94)	20.5 (150)
No	80.6 (1015)	82.2 (434)	79.5 (581)
Primary mode of transport outside of work			
Car	88.6 (1116)	90.7 (479)	87.1 (637)
Walking, public transport or cycling	11.4 (143)	9.3 (49)	12.9 (94)
Smoking status			
Current	2.6 (33)	1.9 (10)	3.1 (23)
Former/never	97.4 (1226)	98.1 (518)	96.9 (708)
Self-rated health			
Very good/excellent/good	89.0 (1121)	88.8 (469)	89.2 (652)
Fair/poor	11.0 (138)	11.2 (59)	10.8 (79)
Home neighbourhood location^b^			
Urban	53.1 (669)	54.9 (290)	51.8 (379)
Rural	46.9 (590)	45.1 (238)	48.2 (352)

^a^Education categories are roughly analogous to high school, college, and university; Percentages (%) may not add to 100.0% due to rounding

^b^ Based on self-reported home postcode address

### Statistical analyses

We calculated descriptive statistics for all of the variables of interest at baseline and follow-up, overall and by sex. Given that the predictors of activity change may differ between men and women, we used sex-stratified linear regression models to estimate the associations between each of the individual and sociodemographic factors at baseline (exposures) and rates of change in the activity measures (outcomes), unadjusted and mutually adjusted for all of the individual/sociodemographic variables of interest, season at baseline and follow-up assessments, wear time at baseline and follow-up, and baseline activity. We adjusted for the individual and sociodemographic factors as assessed at baseline in order to evaluate the role of these factors on change in activity. We expressed the outcomes as rates of change per year (i.e. min/day/year for sedentary time, LPA and MVPA, and counts/minute/year for overall PA) in order to account for variations in follow-up time. Given that wear time could be correlated with measures of activity, we conducted sensitivity analyses additionally adjusting for differences in wear time between baseline and follow-up to ensure that these differences were not responsible for the observed changes in activity. All analyses were based on complete case data and were conducted using Stata/SE 14.1 (College Station, TX: StataCorp LP).

### Findings

Of the 8623 adults who attended the baseline visit and the 5693 adults who attended the follow-up visit, 4169 (48.3%) and 5504 (96.7%) participants, respectively, wore an accelerometer for 7 days following their in-clinic visit. A total of 1813 participants had accelerometer data at both visits. Of these, 154 had invalid data at baseline and/or follow-up (i.e. < 4 valid days of data, data collected at a different epoch lengths, or ≥ 19 h/day of wear-time), leaving complete accelerometer data for 1659 participants. Of these, 1259 participants (75.9%) had complete data on the exposures, outcomes, and covariates of interest. Compliance with the accelerometry protocol was high, with 99% of participants wearing their monitors for at least 12 h/day and 89.5% of participants wearing their monitors between 13 and 16 h per day. There was a small difference in wear-time between baseline and follow-up, with participants at baseline and follow-up wearing their accelerometers for an average of 14.5 (SD = 0.9) and 14.3 h/day (SD = 0.9), respectively (mean difference: 0.16 h/day, 95% CI 0.11, 0.21).

### Descriptive characteristics

The characteristics of the study population at baseline are presented overall, and by sex in Table 1. In brief, participants were on average 67.8 years of age (SD 6.9; Range: 49 to 91) and overweight (mean BMI: 26.4 kg/m^2^, SD = 4.2). The majority had an A-level education or higher, did not have a paid job, and were married or living with a partner. Average follow-up time was 5.7 years (SD = 1.9; Range 1.8 to 9.4 years).

The participants who were included in our analyses were younger, more likely to be educated to an A-level or higher, more likely to have a paid job, more likely to be married/living with a partner, more likely to use a car as a primary mode of transport outside of work, had better self-rated health, and were less sedentary and more active than those who were excluded (Additional file 1: Table S1).

### Accelerometer-assessed activity

**Fig. 1:**
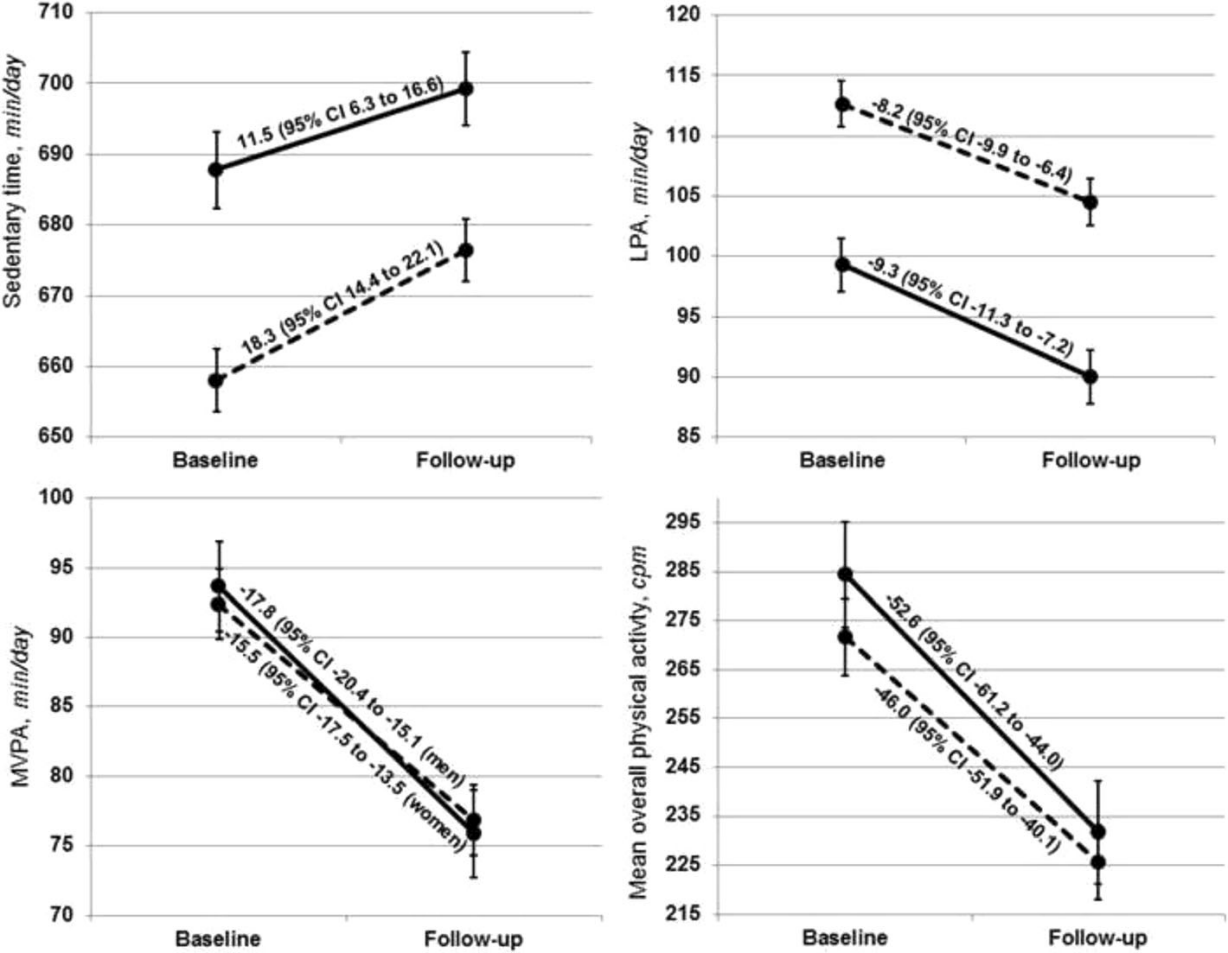
Mean levels of accelerometer-assessed sedentary time, light-intensity physical activity (LPA), moderate-to-vigorous intensity physical activity (MVPA), and overall physical activity (95% confidence intervals) in men and women at baseline and at follow-up with corresponding changes in activity levels (men: bold line; women; dashed line)

At baseline, participants accumulated an average of 11.2 h/day of sedentary time (SD = 1.1), 1.8 h/day of LPA (SD = 0.4), and 1.5 h/day of unbouted MVPA (SD = 0.6). Only 3.7% of participants (6.1% men; 2.1% women) accumulated the recommended level of 150 min/week of MVPA in bouts of at least 10 min. At follow-up, participants accumulated an average of 11.4 h/day of sedentary time (SD = 1.0), 1.6 h/day of LPA (SD = 0.5), and 1.3 h/day of unbouted MVPA (SD = 0.6). Similar to the levels observed at baseline, only 3.7% of participants (4.7% men; 2.9% women) accumulated the recommended level of 150 min/week of MVPA in bouts of at least 10 min at follow-up. Women accumulated 29.7 fewer min/day of sedentary time (95% CI -36.6, − 22.7) and 13.4 more min/day of LPA than men (95% CI 10.4, 16.3) at baseline. These differences were also observed at follow-up, with women accumulating 22.9 fewer min/day of sedentary time (95% CI -29.7, − 16.0) and 14.5 more min/day of LPA than men (95% CI 11.5, 17.4). There were no conclusive differences between men and women in levels of MVPA and overall physical activity at baseline or follow-up (Fig. 1).

### Changes in accelerometer-assessed activity

Sedentary time increased by 3.0 min/day/year (SD = 12.3) and LPA, and unbouted MVPA decreased by 1.7 (SD = 5.4), 3.0 min/day/year (SD = 6.0), respectively. Overall physical activity decreased by 8.8 cpm/year (SD = 18.8). This corresponded to participants increasing the percentage of time they spent in sedentary time by 2.7% (77.1 to 79.8%), and decreasing the amount of time that they spent in LPA and unbouted MVPA by 0.9% (12.3 to 11.4%) and 1.8% (10.6 to 8.8%), respectively. Changes were similar in men and women (Fig. 1), but there was evidence that rates of change varied by age group with rates of change more pronounced in adults ≥65 than in adults < 65 years (Additional file 1: Table S2). For example, MVPA decreased at a rate of 3.7 min/day/year in men ≥65 years but at a rate of 2.2 min/day/year in men < 65 years, representing a difference of − 1.5 min/day/year (95% CI -2.6, − 0.4).

### Correlates of change

Older age and higher BMI were associated with a greater rate of increase in sedentary time and greater rates of decline in LPA, MVPA, and overall physical activity in both men and women. The only exception was for the BMI-MVPA association, which did not appear to be important in women (Table 2). Among men, being married/living with a partner was associated with a 1.8 min/day/year smaller rate of decline in LPA (95% CI 0.5, 3.1), having very good/excellent/good (versus fair or poor) self-reported health was associated with a 1.3 min/day/year smaller rate of decline in LPA (95% CI 0.1, 2.6), being depressed was associated with a greater rate of decline in MVPA (− 1.5 min/day/year, 95% CI -2.7, − 0.2) and overall physical activity (− 5.8 cpm/year; 95% CI -10.4, − 1.3), and relying on a car (as opposed to walking, public transport, or cycling) was associated with 1.6 min/day/year greater rate of decline in MVPA (95% CI -3.2, − 0.03). Among women, being a current smoker was associated with a 3.6 min/day/year greater rate of increase in sedentary time (95% CI 0.04, 7.2), living in an urban neighbourhood was associated with a 2.4 min/day/year greater rate of increase in sedentary time (95% CI 1.0, 3.7), being depressed was associated with a 1.0 min/day/year greater rate of decline in MVPA (95% CI -1.8, − 0.2), being a current smoker was associated with a 8.6 cpm/year greater rate of decline in overall physical activity (95% CI -14.9, − 2.2), and living in an urban neighbourhood was associated with a greater rate of decline in MVPA and overall physical activity (− 1.1 min/day/year, 95% CI -1.9, − 0.3; − 3.3 cpm/year, 95% CI -5.7, − 0.9). In models of MVPA accumulated in bouts ≥10 min (Additional file 1: Table S3), only older age was associated with a greater rate of decline in MVPA in women. Only some of the associations that were observed in the adjusted models (Table 2) were also observed in the unadjusted models (Additional file 1: Table S4).

**Table 2:** Mean differences (95% confidence intervals) in rates of change in accelerometer-assessed sedentary time, light-intensity physical activity (LPA), moderate-to-vigorous intensity physical activity (MVPA), and overall physical activity in men and women, adjusted for all of the covariates of interest, baseline levels of the corresponding activity, season at baseline and follow-up, and wear time at baseline and follow-up^a^ in men (n=528) and women (n=731)

	Sedentary time *(min/day/year)*		LPA *(min/day/year)*		MVPA *(min/day/year)*		Overall physical activity *(counts/minute/year)*	
	Men	Women	Men	Women	Men	Women	Men	Women
Age, *years*	**0.4 (0.2 to 0.5)**	**0.3 (0.2 to 0.5)**	**-0.1 (-0.1 to -0.01)**	**-0.1 (-0.1 to -0.02)**	**-0.3 (-0.3 to -0.2)**	**-0.3 (-0.3 to -0.2)**	**-0.7 (-1.0 to -0.5)**	**-0.8 (-1.1 to -0.6)**
Body mass index, *kg/m*^*2*^	**0.4 (0.1 to 0.6)**	**0.2 (0.1 to 0.3)**	**-0.2 (-0.3 to -0.1)**	**-0.1 (-0.2 to -0.1)**	**-0.2 (-0.3 to -0.05)**	-0.1 (-0.1 to 0.02)	**-0.7 (-1.2 to -0.2)**	**-0.3 (-0.5 to -0.1)**
Education level								
A-level (vs. O-level or lower)	-0.8 (-2.7 to 1.1)	-0.9 (-2.3 to 0.5)	0.4 (-0.5 to 1.3)	0.1 (-0.7 to 0.8)	0.5 (-0.6 to 1.5)	-0.1 (-0.9 to 0.7)	1.3 (-2.5 to 5.0)	-0.1 (-2.5 to 2.4)
Degree (vs. O-level or lower)	-0.004 (-2.4 to 2.4)	-1.6 (-3.4 to 0.2)	-0.7 (-1.9 to 0.4)	-0.3 (-1.3 to 0.7)	0.2 (-1.1 to 1.6)	0.5 (-0.6 to 1.5)	1.5 (-3.3 to 6.2)	1.9 (-1.3 to 5.1)
No paid job	0.5 (-1.5 to 2.6)	-1.2 (-2.8 to 0.4)	-0.2 (-1.1 to 0.8)	0.02 (-0.9 to 0.9)	0.02 (-1.1 to 1.2)	0.5 (-0.5 to 1.5)	-0.8 (-4.9 to 3.2)	1.0 (-2.0 to 4.0)
Married/living with partner	-1.7 (-4.5 to 1.1)	0.7 (-0.9 to 2.2)	**1.8 (0.5 to 3.1)**	0.1 (-0.7 to 1.0)	0.6 (-0.9 to 2.2)	-0.4 (-1.3 to 0.5)	1.2 (-4.3 to 6.7)	-1.0 (-3.7 to 1.8)
Depression	1.7 (-0.6 to 4.0)	0.9 (-0.4 to 2.3)	-0.1 (-1.2 to 1.0)	-0.1 (-0.8 to 0.7)	**-1.5 (-2.7 to -0.2)**	**-1.0 (-1.8 to -0.2)**	**-5.8 (-10.4 to -1.3)**	-2.3 (-4.8 to 0.2)
Dog ownership	-1.6 (-3.7 to 0.6)	-0.2 (-1.8 to 1.3)	1.0 (-0.01 to 2.04)	-0.3 (-1.1 to 0.5)	0.5 (-0.7 to 1.7)	-0.1 (-1.0 to 0.8)	0.9 (-3.4 to 5.2)	1.1 (-1.6 to 3.9)
Car as primary mode of transport outside of work	2.2 (-0.6 to 5.0)	0.5 (-1.3 to 2.4)	-1.3 (-2.7 to 0.0004)	0.03 (-1.0 to 1.0)	**-1.6 (-3.2 to -0.03)**	-0.5 (-1.7 to 0.6)	-4.4 (-10.0 to 1.2)	-1.4 (-4.8 to 2.0)
Current smoker	2.7 (-3.1 to 8.6)	**3.6 (0.04 to 7.2)**	-0.7 (-3.5 to 2.1)	-0.2 (-2.1 to 1.7)	-1.2 (-4.5 to 2.1)	-2.1 (-4.2 to 0.03)	-4.0 (-15.7 to 7.7)	**-8.6 (-14.9 to -2.2)**
Very good/excellent/good self-rated health	-1.1 (-3.7 to 1.5)	-1.0 (-3.1 to 1.0)	**1.3 (0.1 to 2.6)**	0.3 (-0.8 to 1.4)	1.1 (-0.4 to 2.6)	0.5 (-0.7 to 1.7)	0.8 (-4.4 to 6.1)	2.0 (-1.6 to 5.7)
Urban home neighbourhood location	0.5 (-1.2 to 2.2)	**2.4 (1.0 to 3.7)**	-0.03 (-0.8 to 0.8)	-0.2 (-0.9 to 0.5)	0.2 (-0.7 to 1.2)	**-1.1 (-1.9 to -0.3)**	0.7 (-2.7 to 4.1)	**-3.3 (-5.7 to -0.9)**

^a^ Bolded values represent the estimated effects that have at least a small effect based on 95% confidence intervals. Season was coded as a continuous periodic variables at both baseline and follow-up (spring = sin (2 * π * day of year /365.25); winter = cos (2 *π* day of year/365.25)); No paid job versus paid job at present; married/living with partner versus single/widowed/separated/divorced; depressed versus not depressed (based on self-reported depression requiring treatment – yes versus no); car as primary mode of transport outside of work versus walking, public transport or cycling; current smoker versus former/never smoker; very good/excellent/good self-rated health versus fair/poor; urban versus rural home neighbourhood location

## Discussion

Only 3.7% of our sample of older adults met the current MVPA recommendation. Sedentary time increased by 3.0 min/day/year, whereas LPA, unbouted MVPA and overall physical activity decreased by 1.7 min/day/year, 3.0 min/day/year, and 8.8 cpm/year, respectively. Although men accumulated more sedentary time and less LPA than women, there were no conclusive differences between men and women in unbouted MVPA or overall physical activity or in changes in any of the activity measures of interest. There were, however, more pronounced rates of change in activity levels in adults ≥65 than those < 65 years. Correlates of greater rates of increase in sedentary time included older age and higher BMI in men, and older age, higher BMI, smoking, and urban dwelling in women. Correlates of greater rates of decrease in physical activity included older age, higher BMI, living alone, depression, car use, and poor/fair self-rated health car use in men, and older age, higher BMI, depression, smoking, and urban dwelling in women.

Our findings are consistent with those of previous studies in which activity was assessed via questionnaires [10, 11] and accelerometers [6–9]. For example, in an analysis of 3334 participants of the EPIC-Norfolk Study during their transition into retirement, self-reported overall activity, transport-related activity, and occupational-related activity decreased, whereas recreational activity, household-related activity, and TV viewing time increased [10]. Similarly, increases in sedentary time and LPA, and decreases in MVPA were observed over 10 years of follow-up in analyses of 5022 older adults who participated in the English Longitudinal Study of Ageing [11]**.** In this study, correlates of declining physical activity included older age, female sex, having ever smoked, long-standing illness, arthritis, obesity, and depressive symptoms, and lower wealth. In an analysis of 519 community dwelling older adults who participated in the Rush Memory and Aging Project (Chicago, USA), and in whom activity was assessed using accelerometers, total activity declined by approximately 2% per year, with daily activity declining 3% more rapidly for every one-year increase in age and 6% more slowly for every additional year of education [8]. Similarly, in an analysis of 339 community dwelling older adults who participated in the Physical Activity Cohort Study (Tayside, Scotland), total accelerometer-assessed activity declined by 6.2% per year [9].

While previous studies have quantified changes in activity and their correlates, no previous study has, to our knowledge, examined both changes and correlates of these changes in relation to accelerometer-assessed activity volume and a range of activity intensities in both middle-aged and older adults. Our study adds to the current literature by providing an examination of changes during middle and later adulthood in sedentary time, LPA, MVPA, and overall PA and the correlates of these changes. This information may help in the evaluation of interventions and in informing the development of new interventions. For example, we found an annual decrease in MVPA of 3.0 min/day, suggesting a loss of 15 min/day MVPA over a five-year time period. A smaller change in MVPA was observed in the CARDIA study (i.e. a 7.5 min/day decline in MVPA over 10 years of follow-up) [6]. The difference between our finding and the finding from the CARDIA study is likely a result of the CARDIA population being younger. The magnitude of decline that we observed in MVPA is likely to be of clinical significance in our population [31–33]. For example, each additional 10 min/day in MVPA has been associated with a 8% decreased risk of all-cause mortality in older British men after adjustment for age, region of residence, season of accelerometer wear, accelerometer wear time, social class, alcohol use, smoking status, sleep time, living alone status, body mass index, and mobility disability (Hazard Ratio = 0.92, 95% CI 0.86, 0.98) [31].

In terms of informing the development of interventions, our findings demonstrate that changes in activity intensities are comparable between men and women but that rates of change are more pronounced among older adults and that correlates of change appear to be sex-specific. This suggests that intervening on activity changes during older age may need to be a priority and that targeted interventions may be needed to effectively attenuate negative changes in activity in both men and women. We also identified more individual and sociodemographic correlates for unbouted than bouted MVPA. This suggests that mitigating changes in unbouted and bouted MVPA may require intervening on a different set of factors.

Strengths of our study included objective activity monitoring of a large sample of adults over a relatively long period. When interpreting the results, several limitations should also be noted. Firstly, the subset of EPIC-Norfolk participants who were included in our analyses were healthier than the EPIC-Norfolk participants who were excluded. This limits the degree to which our findings can be generalised to other populations, including to the broader EPIC-Norfolk population. Secondly, we aimed to provide an evaluation of the individual and sociodemographic correlates of activity change among older adults. Further research is needed to identify other factors that may influence the trajectory of activity behaviours in later life (e.g. neighbourhood characteristics, attitudes towards activity behaviours) and their interactions. Thirdly, the association observed between smoking and overall physical activity in women should be interpreted with caution; this could have been a spurious finding attributable to the rarity of smoking in our population. Fourthly, we cannot rule out the possibility that there may be rare instances in which the method of identifying non-wear by consecutive zero counts may result in prolonged sedentary time being mistaken for non-wear time.

In conclusion, we demonstrated that sedentary time increases and activity levels decreases over time, with more pronounced rates of change observed in specific sub-groups (e.g. among older and depressed adults). The individual and sociodemographic correlates of activity change appear to be sex-specific. Targeting these correlates in future interventions may help limit increases in sedentary time and decreases in physical activity among English adults.

## Additional file

Additional file 1: **Table S1.** Baseline characteristics of the participants who only attended the baseline visit and participants who attended both the baseline and follow-up visits and had complete accelerometry data at both visits. **Table S2.** Normalised mean rates of change in activity between baseline and follow-up in younger and older men and women with corresponding mean differences between age groups (95% confidence intervals). **Table S3.** Mean differences in rates of change in accelerometer-assessed moderate-to-vigorous intensity physical activity (min/day/year) accumulated in bouts ≥10 min (95% confidence intervals) in men and women, unadjusted and maximally adjusted for all of the covariates of interest, baseline levels of the corresponding activity, season, and baseline and follow-up wear-time.^a^. **Table S4.** Unadjusted mean differences in rates of change in accelerometer-assessed sedentary time, light-intensity physical activity (LPA), moderate-to-vigorous intensity physical activity (MVPA), and overall physical activity (95% confidence intervals) in men and women.^a^ (DOCX 32 kb)
