# Supplementary material for: Descriptive epidemiology of changes in objectively measured sedentary behaviour and physical activity: six-year follow-up of the EPIC-Norfolk cohort
Source: Int J Behav Nutr Phys Act. 2018 Nov 27;15:122. doi: 10.1186/s12966-018-0746-5 (PMC6257971; doi:10.1186/s12966-018-0746-5)
Supplement: Supplementary file 1 — Table S1. Baseline characteristics of the participants who only attended the baseline visit and participants who attended both the baseline and follow-up visits and had complete accelerometry data at both visits. Table S2. Normalised mean rates of change in activity between baseline and follow-up in younger and older men and women with corresponding mean differences between age groups (95% confidence intervals). Table S3. Mean differences in rates of change in accelerometer-assessed moderate-to-vigorous intensity physical activity (min/day/year) accumulated in bouts ≥10 min (95% confidence intervals) in men and women, unadjusted and maximally adjusted for all of the covariates of interest, baseline levels of the corresponding activity, season, and baseline and follow-up wear-time.a. Table S4. Unadjusted mean differences in rates of change in accelerometer-assessed sedentary time, light-intensity physical activity (LPA), moderate-to-vigorous intensity physical activity (MVPA), and overall physical activity (95% confidence intervals) in men and women.a (DOCX 32 kb) [file 12966_2018_746_MOESM1_ESM.docx]

**Additional file 1**

| **Table S1.** Baseline characteristics of the participants who only attended the baseline visit and participants who attended both the baseline and follow-up visits and had complete accelerometry data at both visits. | | |
| --- | --- | --- |
|  | **Only Baseline Visit Attended**^a^ | **Both Baseline & Follow-up Visits Attended** |
|  | *n=4,558* | *n=1,259* |
|  | **mean (SD)** | |
| Age, *years* | 70.5 (8.3) | 67.8 (6.9) |
| Body mass index, *kg/m^2^* | 27.0 (4.4) | 26.4 (4.2) |
|  | **% (n)** | |
| Education level |  |  |
| *O-level or lower* | 40.8 (1,860) | 34.4 (433) |
| *A-level* | 43.5 (1,981) | 47.2 (594) |
| *Degree* | 15.7 (715) | 18.4 (232) |
| Paid job at present |  |  |
| *Yes* | 22.0 (973) | 28.4 (357) |
| *No* | 78.0 (3,454) | 71.6 (902) |
| Marital status |  |  |
| *Married/living with partner* | 75.8 (3,341) | 83.1 (1,046) |
| *Single/widowed/separated/divorced* | 24.2 (1,069) | 16.9 (213) |
| Self-reported depression requiring treatment |  |  |
| *Yes* | 21.2 (967) | 21.7 (273) |
| *No* | 78.8 (3,591) | 78.3 (986) |
| Household dog ownership |  |  |
| *Yes* | 17.5 (570) | 19.4 (244) |
| *No* | 82.5 (2,690) | 80.6 (1,015) |
| Primary mode of transport outside of work |  |  |
| *Car* | 83.9 (2,776) | 88.6 (1,116) |
| *Walking, public transport or cycling* | 16.1 (533) | 11.4 (143) |
| Smoking status |  |  |
| *Current* | 4.7 (211) | 2.6 (33) |
| *Former/never* | 95.3 (4,243) | 97.4 (1,226) |
| Self-rated health |  |  |
| *Very good/excellent/good* | 81.1 (3,578) | 89.0 (1,121) |
| *Fair/poor* | 18.9 (836) | 11.0 (138) |
| Home neighbourhood location |  |  |
| *Urban* | 52.8 (1,705) | 53.1 (669) |
| *Rural* | 47.2 (1,526) | 46.9 (590) |
| MVPA recommendations |  |  |
| *≥150 min/week (in bouts of ≥10 min)* | 2.3 (49) | 3.7 (47) |
| *<150 min/week (in bouts of ≥10 min)* | 97.7 (2,101) | 96.3 (1,212) |
|  | **mean (SD)** | |
| Sedentary time, *min/day* | 677.6 (68.6) | 670.6 (63.8) |
| LPA, *min/day* | 100.6 (28.0) | 107.1 (27.0) |
| MVPA, *min/day* | 79.9 (37.6) | 92.9 (36.1) |
| Overall PA, *counts per minute* | 237.7 (116.3) | 277.0 (115.9) |

^a^ Body mass index (n=4,540); education (n=4,556); paid job at present (n=4,427); marital status (n=4,410); dog ownership (n=3,260); car use (n=3,309); smoking (n=4,454); self-rated health (n=4,414); home neighbourhood location (n=3,231); MVPA recommendations, sedentary time, LPA, MVPA, overall physical activity (n=2,150)

| **Table S2**. Normalised mean rates of change in activity between baseline and follow-up in younger and older men and women with corresponding mean differences between age groups (95 % confidence intervals). | | | |
| --- | --- | --- | --- |
|  | **Men** | | **Mean differences between age groups** |
|  | **<65 years** | **≥65 years** |  |
|  | *n=169* | *n=359* |  |
| Sedentary time, min/day/yr | 0.5 (-1.2 to 2.3) | 3.1 (1.7 to 4.6) | 2.6 (0.2 to 5.1) |
| LPA, min/day/yr | -1.5 (-2.4 to -0.7) | -2.0 (-2.6 to -1.5) | -0.5 (-1.5 to 0.5) |
| MVPA, min/day/yr | -2.2 (-3.0 to -1.3) | -3.7 (-4.4 to -3.0) | **-1.5 (-2.6 to -0.4)** |
| Overall PA, cpm/yr | -6.0 (-9.3 to -2.8) | -10.6 (-12.7 to -8.5) | **-4.6 (-8.3 to -0.8)** |
|  | **Women** | |  |
|  | **<65 years** | **≥65 years** |  |
|  | *n=319* | *n=412* |  |
| Sedentary time, min/day/yr | 3.1 (1.9 to 4.3) | 3.8 (2.7 to 5.0) | 0.7 (-0.9 to 2.4) |
| LPA, min/day/yr | -1.1 (-1.6 to -0.6) | -1.9 (-2.5 to -1.4) | **-0.8 (-1.6 to -0.03)** |
| MVPA, min/day/yr | -2.2 (-2.9 to -1.6) | -3.4 (-4.0 to -2.8) | **-1.2 (-2.0 to -0.3)** |
| Overall PA, cpm/yr | -6.8 (-8.7 to -4.8) | -10.1 (-11.7 to -8.4) | **-3.3 (-5.8 to -0.8)** |

| **Table S3.** Mean differences in rates of change in accelerometer-assessed moderate-to-vigorous intensity physical activity (min/day/year) accumulated in bouts ≥10 minutes (95% confidence intervals) in men and women, unadjusted and maximally adjusted for all of the covariates of interest, baseline levels of the corresponding activity, season, and baseline and follow-up wear-time.^a^ | | | | |
| --- | --- | --- | --- | --- |
|  | **Unadjusted univariate models** | | **Maximally adjusted model** | |
|  | **Men**  *(n=528)* | **Women**  *(n=731)* | **Men**  *(n=528)* | **Women**  *(n=731)* |
| Age, *years* | -0.001 (-0.03 to 0.02) | -0.01 (-0.02 to 0.01) | -0.02 (-0.04 to 0.01) | **-0.02 (-0.04 to -0.002)** |
| Body mass index, *kg/m^2^* | 0.01 (-0.04 to 0.1) | 0.01 (-0.01 to 0.03) | -0.02 (-0.07 to 0.03) | -0.01 (-0.04 to 0.01) |
| Education level |  |  |  |  |
| *A-level (vs. O-level or lower)* | 0.02 (-0.4 to 0.4) | -0.1 (-0.4 to 0.1) | 0.04 (-0.3 to 0.4) | -0.01 (-0.2 to 0.2) |
| *Degree (vs. O-level or lower)* | -0.4 (-0.9 to 0.1) | 0.1 (-0.2 to 0.4) | -0.03 (-0.5 to 0.5) | 0.2 (-0.1 to 0.5) |
| No paid job | -0.2 (-0.5 to 0.2) | -0.1 (-0.4 to 0.1) | 0.1 (-0.4 to 0.5) | 0.04 (-0.2 to 0.3) |
| Married/living with partner | 0.1 (-0.5 to 0.7) | -0.1 (-0.4 to 0.2) | 0.1 (-0.5 to 0.7) | -0.1 (-0.4 to 0.1) |
| Depression | -0.5 (-0.9 to 0.02) | 0.1 (-0.2 to 0.3) | -0.4 (-0.8 to 0.1) | 0.1 (-0.1 to 0.3) |
| Dog ownership | 0.1 (-0.3 to 0.6) | 0.02 (-0.3 to 0.3) | 0.1 (-0.3 to 0.5) | -0.04 (-0.3 to 0.2) |
| Car as primary mode of transport outside of work | -0.2 (-0.7 to 0.4) | -0.1 (-0.5 to 0.2) | -0.5 (-1.1 to 0.02) | -0.2 (-0.5 to 0.1) |
| Current smoker | -0.3 (-1.5 to 1.0) | -0.2 (-0.8 to 0.5) | -0.5 (-1.7 to 0.6) | -0.5 (-1.1 to 0.1) |
| Very good/excellent/good self-rated health | -0.2 (-0.7 to 0.4) | -0.3 (-0.6 to 0.1) | -0.03 (-0.6 to 0.5) | -0.1 (-0.4 to 0.2) |
| Urban home neighbourhood location | 0.03 (-0.3 to 0.4) | -0.1 (-0.3 to 0.1) | 0.1 (-0.2 to 0.5) | -0.1 (-0.4 to 0.1) |

**^a^** Bolded values represent the estimated effects that have at least a small effect based on 95% confidence intervals. Season was coded as a continuous periodic variables at both baseline and follow-up (spring = sin (2 * π * day of year /365.25); winter = cos (2 *π* day of year/365.25)); No paid job *versus* paid job at present; married/living with partner *versus* single/widowed/separated/divorced; depressed *versus* not depressed (based on self-reported depression requiring treatment – yes *versus* no); car as primary mode of transport outside of work *versus* walking, public transport or cycling; current smoker *versus* former/never smoker; very good/excellent/good self-rated health *versus* fair/poor; urban *versus* rural home neighbourhood location.

| **Table S4.** Unadjusted mean differences in rates of change in accelerometer-assessed sedentary time, light-intensity physical activity (LPA), moderate-to-vigorous intensity physical activity (MVPA), and overall physical activity (95% confidence intervals) in men and women.^a^ | | | | |
| --- | --- | --- | --- | --- |
|  | **Sedentary time** (*min/day/year)* | | **LPA** (*min/day/year)* | |
|  | **Men**  *(n=528)* | **Women**  *(n=731)* | **Men**  *(n=528)* | **Women**  *(n=731)* |
| Age, *years* | 0.2 (0.1 to 0.4) | 0.03 (-0.1 to 0.2) | **-0.1 (-0.2 to -0.03)** | **-0.1 (-0.2 to -0.04)** |
| Body mass index, *kg/m^2^* | -0.1 (-0.4 to 0.3) | 0.004 (-0.2 to 0.2) | **-0.2 (-0.3 to -0.04)** | **-0.1 (-0.2 to -0.03)** |
| Education level |  |  |  |  |
| *A-level (vs. O-level or lower)* | -1.5 (-4.2 to 1.1) | -1.3 (-3.1 to 0.6) | 0.2 (-0.8 to 1.3) | 0.3 (-0.5 to 1.2) |
| *Degree (vs. O-level or lower)* | -2.6 (-6.0 to 0.7) | -2.3 (-4.7 to 0.1) | 0.2 (-1.2 to 1.5) | 1.0 (-0.2 to 2.1) |
| No paid job | **3.6 (1.2 to 6.1)** | 1.6 (-0.3 to 3.5) | -0.5 (-1.5 to 0.5) | -0.2 (-1.1 to 0.7) |
| Married/living with partner | -1.1 (-4.9 to 2.8) | 0.3 (-1.7 to 2.4) | 0.4 (-1.1 to 2.0) | 0.1 (-0.9 to 1.0) |
| Depression | 2.1 (-1.1 to 5.4) | 1.0 (-0.9 to 2.9) | 0.3 (-1.0 to 1.6) | 0.1 (-0.7 to 1.0) |
| Dog ownership | 1.1 (-1.9 to 4.0) | 1.2 (-0.8 to 3.3) | 0.5 (-0.7 to 1.7) | **-1.0 (-2.0 to -0.04)** |
| Car as primary mode of transport outside of work | -0.3 (-4.2 to 3.6) | -1.5 (-4.0 to 1.0) | -1.4 (-3.0 to 0.2) | 0.1 (-1.0 to 1.3) |
| Current smoker | -1.9 (-10.3 to 6.5) | 2.5 (-2.2 to 7.3) | 0.2 (-3.2 to 3.5) | 1.5 (-0.8 to 3.7) |
| Very good/excellent/good self-rated health | -0.8 (-4.4 to 2.8) | 0.8 (-1.9 to 3.5) | 0.8 (-0.7 to 2.2) | 1.0 (-0.3 to 2.3) |
| Urban home neighbourhood location | -1.4 (-3.7 to 0.9) | 1.1 (-0.6 to 2.7) | 0.5 (-0.4 to 1.4) | 0.6 (-0.2 to 1.4) |
|  | **MVPA** (*min/day/year)* | | **Overall physical activity** *(cpm/year)* | |
|  | **Men**  *(n=528)* | **Women**  *(n=731)* | **Men**  *(n=528)* | **Women**  *(n=731)* |
| Age, *years* | **-0.1 (-0.2 to -0.05)** | **-0.1 (-0.2 to -0.1)** | **-0.3 (-0.6 to -0.1)** | **-0.3 (-0.5 to -0.1)** |
| Body mass index, *kg/m^2^* | -0.5 (-0.2 to 0.1) | 0.003 (-0.1 to 0.1) | -0.005 (-0.5 to 0.5) | 0.1 (-0.2 to 0.4) |
| Education level |  |  |  |  |
| *A-level (vs. O-level or lower)* | 0.8 (-0.4 to 2.1) | -0.2 (-1.1 to 0.7) | 2.3 (-1.8 to 6.4) | -1.0 (-3.8 to 1.8) |
| *Degree (vs. O-level or lower)* | 1.0 (-0.6 to 2.6) | 1.0 (-0.2 to 2.2) | 1.8 (-3.4 to 6.9) | 2.1 (-1.6 to 5.7) |
| No paid job | -0.8 (-1.9 to 0.4) | -0.6 (-1.6 to 0.4) | -3.5 (-7.3 to 0.2) | -2.3 (-5.2 to 0.6) |
| Married/living with partner | -1.2 (-3.0 to 0.6) | 0.4 (-0.7 to 1.4) | -1.7 (-7.7 to 4.3) | 1.4 (-1.6 to 4.5) |
| Depression | -1.2 (-2.7 to 0.4) | -0.6 (-1.5 to 0.4) | **-5.5 (-10.5 to -0.5)** | -1.4 (-4.3 to 1.4) |
| Dog ownership | **-1.4 (-2.8 to -0.1)** | **-1.3 (-2.3 to -0.2)** | -4.1 (-8.6 to 0.5) | -2.2 (-5.3 to 1.0) |
| Car as primary mode of transport outside of work | -1.0 (-2.9 to 0.8) | -0.02 (-1.3 to 1.3) | -0.8 (-6.8 to 5.3) | 1.3 (-2.5 to 5.1) |
| Current smoker | 1.1 (-2.8 to 5.0) | -0.2 (-2.6 to 2.2) | 2.4 (-10.5 to 15.3) | -2.9 (-10.2 to 4.4) |
| Very good/excellent/good self-rated health | -0.03 (-1.7 to 1.7) | 0.04 (-1.3 to 1.4) | -1.9 (-7.5 to 3.7) | -0.8 (-4.9 to 3.3) |
| Urban home neighbourhood location | 0.7 (-0.4 to 1.8) | -0.1 (-1.0 to 0.7) | 1.3 (-2.2 to 4.8) | -0.7 (-3.3 to 1.8) |

**^a^** Bolded values represent the estimated effects that have at least a small effect based on 95% confidence intervals. No paid job *versus* paid job at present; married/living with partner *versus* single/widowed/separated/divorced; depressed *versus* not depressed (based on self-reported depression requiring treatment); car as primary mode of transport outside of work *versus* walking, public transport or cycling; current smoker *versus* former/never smoker; very good/excellent/good self-rated health *versus* fair/poor; urban *versus* rural home neighbourhood location.
